# Familial Breast Cancer: Disease Related Gene Mutations and Screening Strategies for Chinese Population

**DOI:** 10.3389/fonc.2021.740227

**Published:** 2021-12-01

**Authors:** Lu Shen, Shizhen Zhang, Kaiyue Wang, Xiaochen Wang

**Affiliations:** Department of Breast Surgery and Oncology, Key Laboratory of Cancer Prevention and Intervention, Ministry of Education, The Second Affiliated Hospital, Zhejiang University School of Medicine, Hangzhou, China

**Keywords:** family history, familial breast cancer, gene mutations, genetic syndromes, screening, genetic counseling

## Abstract

**Background:**

About 5%–10% of the breast cancer cases have a hereditary background, and this subset is referred to as familial breast cancer (FBC). In this review, we summarize the susceptibility genes and genetic syndromes associated with FBC and discuss the FBC screening and high-risk patient consulting strategies for the Chinese population.

**Methods:**

We searched the PubMed database for articles published between January 2000 and August 2021. Finally, 380 pieces of literature addressing the genes and genetic syndromes related to FBC were included and reviewed.

**Results:**

We identified 16 FBC-related genes and divided them into three types (high-, medium-, and low-penetrance) of genes according to their relative risk ratios. In addition, six genetic syndromes were found to be associated with FBC. We then summarized the currently available screening strategies for FBC and discussed those available for high-risk Chinese populations.

**Conclusion:**

Multiple gene mutations and genetic disorders are closely related to FBC. The National Comprehensive Cancer Network (NCCN) guidelines recommend corresponding screening strategies for these genetic diseases. However, such guidelines for the Chinese population are still lacking. For screening high-risk groups in the Chinese population, genetic testing is recommended after genetic counseling.

## 1 Introduction

As reported by the 2020 Cancer Statistics, the most common type of cancer diagnosed is breast cancer (BC), with approximately 2.26 million new cases worldwide in 2020. In China, BC is the fourth most commonly diagnosed malignancy (approximately 420,000 patients in 2020), after lung cancer, colorectal cancer, and stomach cancer. Notably, BC is the most commonly diagnosed cancer and the leading cause of death among women (1). BCs with a hereditary background are termed familial breast cancers (FBCs) and receive significant focus because they make up about 5%–7% of the BCs (2, 3). Many susceptibility genes, such as *BRCA1/2*, have been found to be related to FBC (4). Moreover, several genetic syndromes, such as hereditary breast and ovarian cancer (HBOC) syndrome, have also been associated with FBC (5). Due to the substantial heterogeneity among patients with BC, the prevalence and genetic susceptibility of BC in different races or regions vary depending on the type of disease. At present, several disease-related gene mutations have been confirmed in FBCs, and some exist specifically in the Chinese population.

In this review, we aim to summarize the FBC-related susceptibility genes and syndromes, introduce risk assessment models and explore screening methods, such as genetic counseling, for Chinese individuals with a potentially high risk of FBC.

## 2 Methods

**Figure 1:**
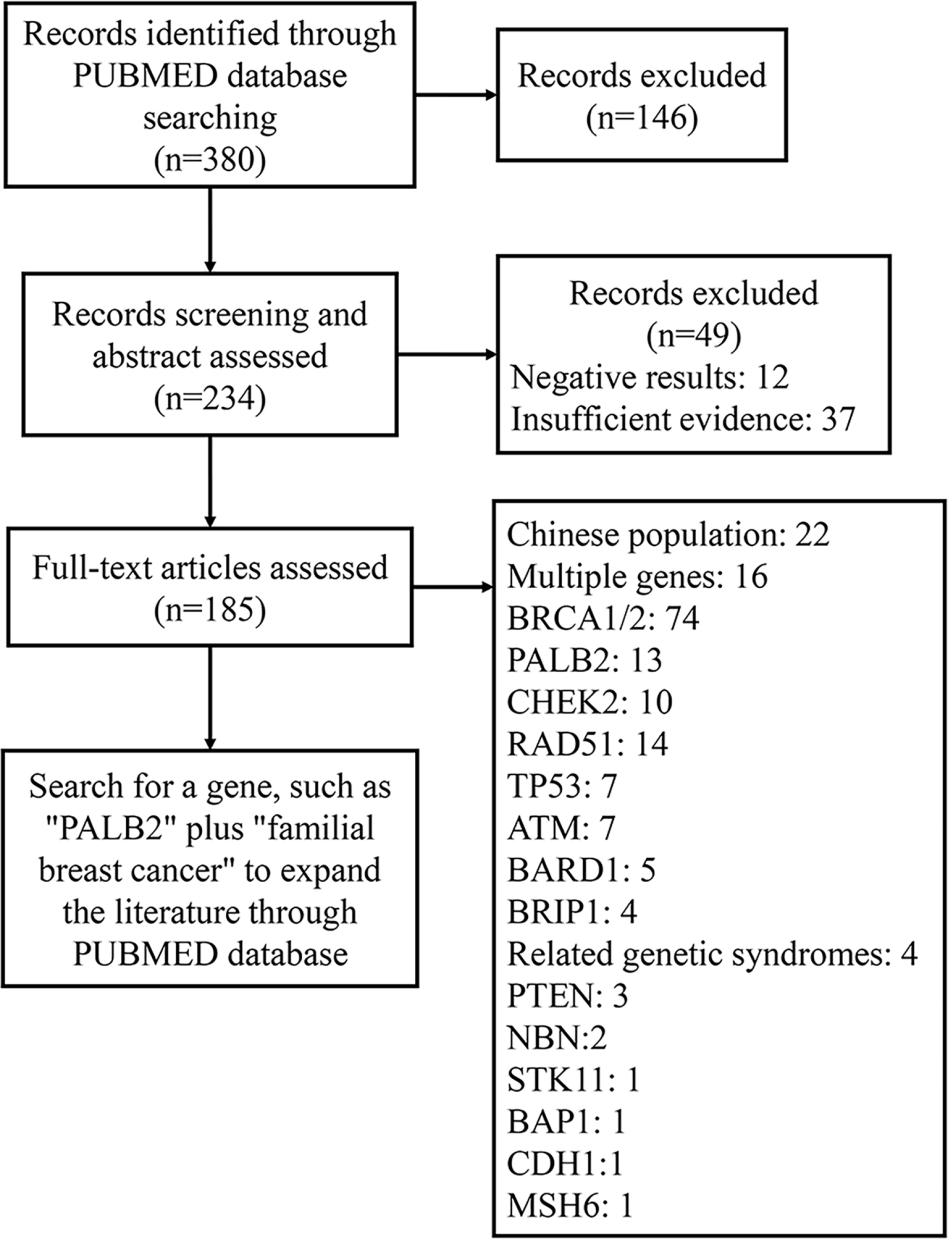
Literature screening steps.

We searched the literature published in PubMed between January 2000 and August 2021 by searching the terms “familial breast cancer,” “family breast cancer,” and “gene” in the title or abstract. Subsequently, 380 studies addressing genetic mutations in familial breast cancer were identified. Among those, we excluded irrelevant articles and classified the remaining articles (**Figure 1**). After analyzing the selected articles, we selected the most relevant genes and searched the PubMed database to acquire pertinent information.

## 3 Results

### 3.1 Familial Breast Cancer (FBC)

**Figure 2:**
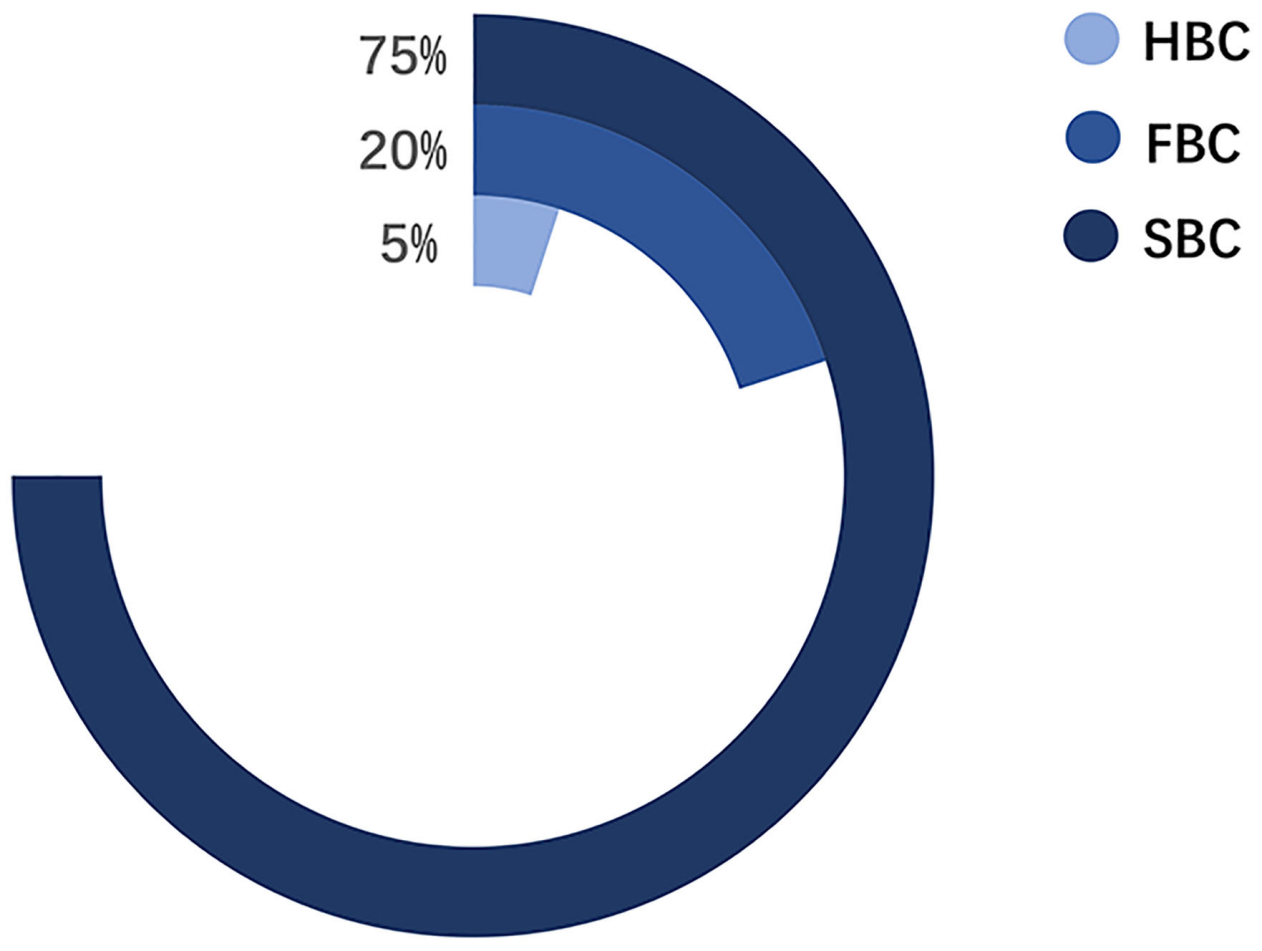
The proportion of three categories in BC.

Pathologically, BCs are classified into four subtypes: Luminal A, Luminal B, HER2-positive, and triple-negative BC. The treatment and prognosis of each subtype differ (6). Epidemiologically, BCs are mainly divided into three categories: 1) sporadic breast cancer (SBC), 2) hereditary breast cancer (HBC), and 3) FBC (**Figure 2**) (7). HBC differs from FBC and refers explicitly to patients with BC with definite genetic factors, accounting for approximately 5%–10% of the BCs. About 10%–15% of the HBCs have a positive family history (FH) (8, 9). Meanwhile, FBC is a subset of BC within a family, where the underlying genetic cause is not entirely known. The incidence of BC within a family is mainly due to genetic factors and partly due to environmental factors (10, 11). Environmental factors come from both shared and non-shared environments. The shared environment includes eating habits and lifestyles. The non-shared environment includes age at menarche, age at first full-term pregnancy, and age at menopause (10).. According to the prediction model proposed by Lichtenstein et al., the shared environmental factors contributed 6%, and non-shared environmental factors contributed 67% of the risk for developing BC (12). In subsequent studies, the discovery of low-penetrance genes reduced the influence rate of environmental factors to 27% (11). Recent studies have focused on the contribution of gene-environment interactions in familial aggregation (13). Nevertheless, environmental factors are still a major cause of FBC in various members of a family.

The first typical case of FBC was reported by Broca in 1866. In that case, ten females were diagnosed with BC among the thirty-eight family members, which strongly suggested that the family members carried specific BC susceptibility genes or were exposed to the same environmental factors contributing to BC. In 1979, Lynch defined FBC with clinical characteristics of earlier onset age, two or more first-degree relatives with a history of BC, a higher incidence of bilateral BC, and multicentric cancer (14). In 1988, Phipps revised the definition of FBC to include early age of onset, excess bilaterality, specific tumor association with colon and ovary, and vertical transmission (15). Presently, the criteria for FBC diagnosis include (1) in addition to the first patient (proband) in the family, there are more than three BCs in first-degree relatives or: (2) in addition to the proband in a family, there are more than 2 BCs in first-degree relatives, and at least one of them meets one of the following conditions: age less than 40 years at the time of onset, simultaneous or heterochronous bilateral BC, or simultaneous or heterochronous non-breast repeat cancer (16).

The definitions of FBC and HBC are unclear and partially overlapping. Many studies have not distinguished between FBC and HBC in terms of clinical features and prognosis (17). One important reason for distinguishing FBC from other types of BCs is that it allows us to target the high-risk population and predict the risk of BC based on FH. As early as 1972, Anderson reported that first-degree relatives of patients with BC have a 2–3 times higher risk of developing BC than those with no FH of BC; if the patient has bilateral BC, the risk is about five times higher for the relatives and if the patient has premenopausal bilateral BC, the risk increases to 9 times higher for relatives (18). Recent studies have found that as the number of first-degree relatives with BC increases, the probability that women 20–80 years old with an FH of BC may develop BC increases correspondingly (19). Interestingly, a positive FH of BC appears to increase the patient’s lifetime risk of FBC because FH may affect their BC screening behaviors (20). With the combination of molecular genetic techniques and knowledge of FH of BC, genetic counselors can provide more precise disease risk prediction, which is critical for the prevention and treatment of FBC.

### 3.2 Gene Mutations Related to FBC

**Figure 3:**
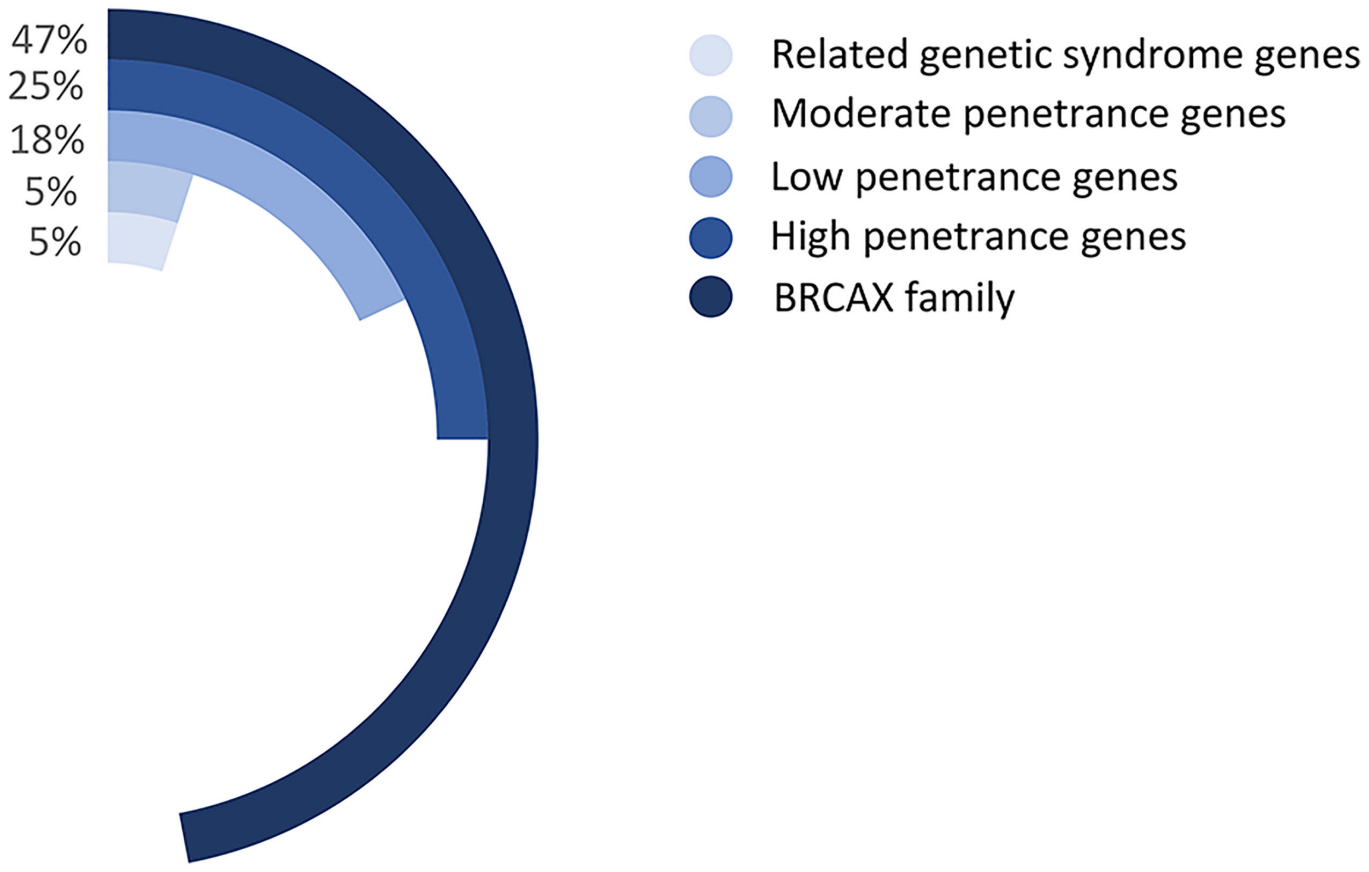
The proportion of various gene mutations in FBCs. Related genetic syndrome genes refer to mutated genes found in genetic syndromes related to BC, such as HBOC. Related genetic syndrome genes accounts for about 5% of whole mutation genes. Low-, moderate-, and high- penetrance genes are classified as BC mutation genes based on lifetime risk of disease. The BRCAX family accounted for the largest proportion, reaching 47% of whole mutation genes. Patients belong to The BRCAX family have not found any currently known disease-causing mutation genes.

Multiple genes have been identified to be associated with FBC. For example, *BRCA1/2* gene mutations account for 5% of the BC mutant genes and can lead to 16% to 25% of the FBC cases (21, 22). Moreover, mutant genes associated with genetic syndromes, such as *TP53*, *PTEN*, *STK11*, and *CDH1*, account for 5% of the FBC risk. Moderate mutations in penetrance genes, such as *ATM* gene mutations, also account for approximately 5% of the risk of FBC. More than 180 low-sensitivity genes account for approximately 18% of the FBC risk (23). However, the remaining FBCs showed no mutations in any of these genes and were therefore classified as *BRCAX* (*BRCA1/2*-negative, high-risk) BCs (**Figure 3**). This type of FBC may carry one or multiple unidentified genetic mutations (22, 24).

The gene penetration rate, which refers to the estimated risk of a specific disease in the presence of this genotype of a particular gene, is commonly used as a genetic biomarker to predict cancer risk. Relative risk (RR) represents the risk of obtaining a disease compared with the general population’s risk. In general, BC susceptibility genes are associated with different risk levels and are roughly divided into high- (RR ≥5.0), moderate- (1.5≤ RR <5.0), and low-penetrance (1.0≤ RR <1.5) alleles (25, 26) (**Table 1**).

**Table 1:** Penetrance of genes in BC.

Penetrance	Gene
High Penetrance (rare)	*BRCA1/BRCA2*
(Related Genetic Syndrome)^1^	*PALB2/FANCN* *TP53* *PTEN* *CDH1* *STK11*
Moderate Penetrance (uncommon)	*NF1* *CHEK2* *ATM* *NBN* *RAD51C/D* *MLH1*
Low Penetrance (common)	*MSH2* *MSH6*

^1^TP53, PTEN, CDH1, STK11 belong to the category of high penetrance genes and are related to genetic syndromes.

People with high-penetrance alleles usually have a lifetime risk of developing BC of more than 50%, those with moderate-penetrance have a lifetime risk greater than 20%, and those with low-penetrance alleles have a lifetime risk of 10%–20% (27–29). Thus, discovering the mutant genes highly related to BC is vital for disease screening and prediction.

### 3.3 Genes With High Penetrance

#### 3.3.1 *BRCA1* and *BRCA2*

**Figure 4:**
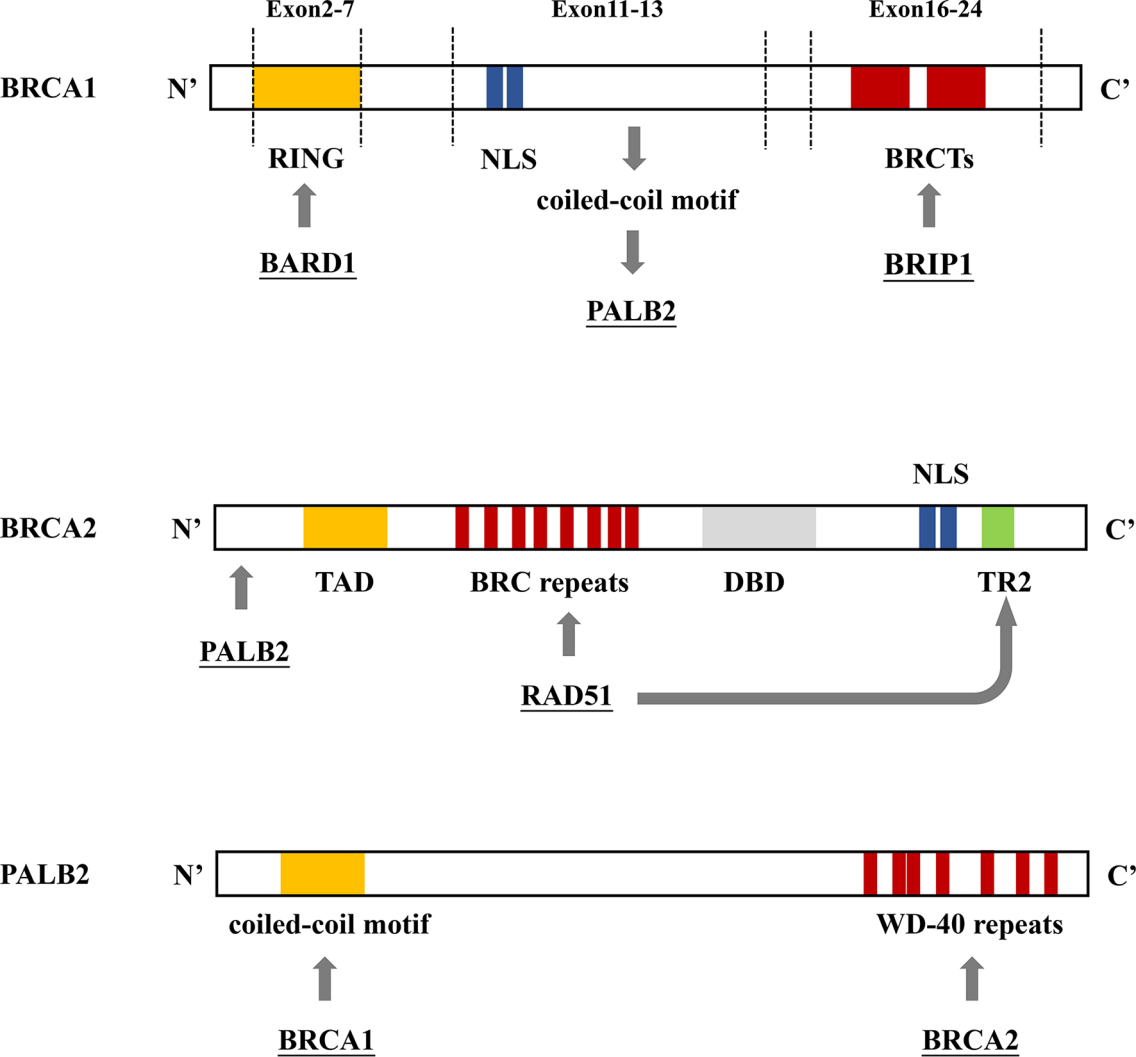
*BRCA1/2* and *PALB2* gene binding sites. **(A)** *BRCA1* contains 3 mutation domains: a central N-terminal RING fingerprint domain (exons 2–7) that binds to BARD, two nuclear localization signals (NLSs) (exons 11–13) that import *BRCA1* into the nucleus, and a C-terminal BRCT domain (exons 16–24) that interact with *BRIP1*. **(B)** The N-terminal region of *BRCA2* interacts with *PALB2*. The N-terminal contains the topologically associating domain (TAD). Eight *RAD51* binding sites (BRC repeats) located on the central part and TR2 located on the C-terminal bind to *RAD51* to promote the *RAD51*-mediated DNA strand exchange process. The C-terminal contains the DNA binding domain (DBD), which includes 3 oligonucleotide/oligosaccharide-binding folds that bind to double-stranded DNA, two NLSs. **(C)** The N-terminal coiled-coil motif of *PALB2* binds to *BRCA1*; the C-terminal WD-40 repeats bind to *BRCA2*, forming the *BRCA1/PALB2/BRCA2* complex.

**Figure 5:**
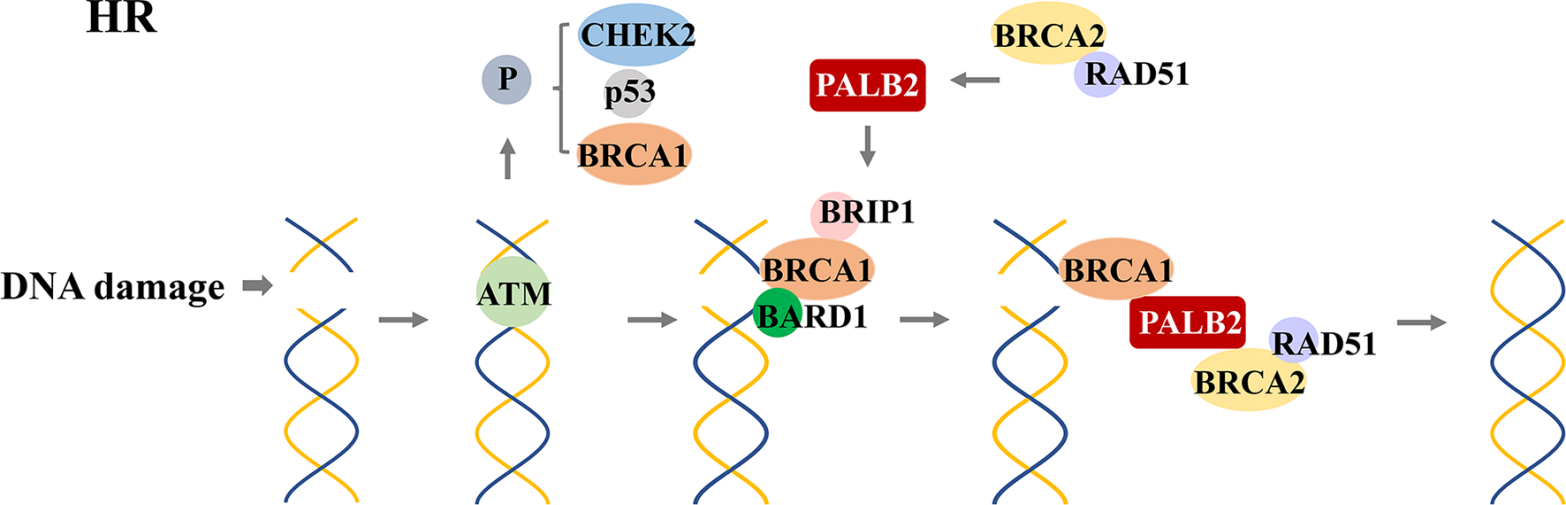
*RAD51*-mediated homologous recombination (HR) for DNA repair. After detecting DNA double-strand damage, *ATM* is recruited and activated, leading to the phosphorylation of downstream effectors, including *BRCA1*, *p53*, and *CHEK2*. After phosphorylation, *BRCA1* and *BRAD1* form a heterodimer; *BRIP1* interacts with BRCT repeats; which constitute a scaffold to recruit *BRCA2*, *PALB2*, and *RAD51* to form a complex. This complex locates the DNA damage site and promotes the HR process.

In 1990, chromosome 17q21.2 was identified to be related to FBC (30), and the loss of 17q heterozygosity is frequently detected in familial breast and ovarian tumors (31). Using positional cloning, the BC susceptibility gene *BRCA1* was found to be located on chromosome 17 at the 17q21 position (32). In 1995, the *BRCA2* gene was identified on chromosome 13q12.36 (7). *BRCA1* contains 24 exons and encodes a protein of 1,863 amino acids. The exon contains three mutation domains: a central N-terminal RING fingerprint domain (exons 2–7), a C-terminal BRCT domain (exons 16–24), and exons 11–13. The N-terminal RING fingerprint domain of *BRCA1* binds to *BRCA1*-associated RING domain protein 1 (BARD1) (33), and the C-terminal BRCT domain binds to the phosphorylated protein (34, 35). *BRCA2* contains 27 exons and encodes a protein of 3,418 amino acids. The N-terminal of *BRCA2* contains the transcriptional activation domain, the middle section includes eight conserved motifs called BRC repeats that bind to *RAD51*, and the C-terminal contains the DNA-binding domain, two nuclear localization signals, and one TR2 (C-terminal *RAD51* binding site) (36). *BRCA1* and *BRCA2* participate in *RAD51*-mediated homologous recombination (HR) for DNA repair. In the case of DNA double-strand damage, *BRCA1* can be accurately located and phosphorylated at the damage site, and *BRCA2* forms a complex with *RAD51* to move it from the site of synthesis to the site of DNA damage processing (37). Furthermore, *PALB2* acts as a bridge between *BRCA1* and *BRCA2*. Its N-terminal coiled-coil motif binds to the coiled-coil motif encoded by exon 11 of *BRCA1*, and its C-terminal WD-40 repeats bind to the N-terminal of *BRCA2* to form the *BRCA1/PALB2/BRCA2* complex (**Figures 4**, **5**). This complex is critical for HR after DNA double-strand breaks (38). In the absence of *BRCA1* and *BRCA2*, HR is suppressed. DNA damage repair is alternatively carried out through the non-homologous end-joining pathway, which is more error-prone and leads to genome instability (7, 39). As a result, *BRCA1* and *BRCA2* play an essential role in maintaining genome integrity (40).

As tumor suppressor genes, mutations of *BRCA1* (MIM 113705) and *BRCA2* (MIM 600185) are closely related to the development of BC (41, 42). Carriers of these mutations have a 10–20 times higher risk of developing BC than those without *BRCA* mutations (43). Approximately 16%–25% of the FBC cases harbor harmful variants of *BRCA1* and *BRCA2* (44, 45). Although many genetic variants of *BRCA1* and *BRCA2* have been recorded, approximately 53%–55% of the variants occur in only one family. The most common *BRCA1* variants were 185delAG (16.5%), 5382insC (8.8%), and missense variant C61G (1.8%). Meanwhile, the most frequently reported *BRCA2* variants include 6174delT (9.6%), K3326X (2.6%), 3036del4 (0.9%), and 6503delTT (0.8%) (46). Notably, the mutation spectrum of *BRCA1*/*BRCA2* varies significantly depending on geographic origin or ethnicity (47). For example, in China, the mutation rate of *BRCA2* in FBC was higher than that of *BRCA1* in the Shandong Province (48). However, other studies including different Chinese regions, such as Shanghai and the Henan Province, showed that the mutation rate of *BRCA1* in FBC is higher than that of *BRCA2* (49). The variant hotspots of *BRCA1* in the Henan cohort were A3113G and A3780G, which was first reported in this population (50). Moreover, in the participants from Shanghai, two other new splice site variants in the *BRCA1* gene (IVS17-1G>T, IVS21+1G>C) were discovered (51). *BRCA2* gene mutations dominated in FBCs of the eastern Shandong population, and three *BRCA2* gene variants, 2001delTTAT, 4099C to T, and 5873C to A, were discovered for the first time in this population (52). A new *BRCA1* missense variant, c.5191C>A, was identified in the Taiwanese population, but whether it is pathogenic remains inconclusive (53). Moreover, the recurrent variant of *BRCA1*, 1100delAT, was found in the Shanghai, Jinan, Qingdao, and Shenyang populations (54), while the *BRCA1* c.470_471delCT and c.981_982delAT variants were considered to be recurrent variants in the Hong Kong population (55). Additionally, racial differences have an impact on gene mutations. For example, a Singapore study that studied individuals with a personal or FH of familial breast/ovarian cancers (FBOCs) with the *BRCA1* c.442-22_442-13del variant, found that this variant was more common in patients of Chinese origin. The study also implied that the *BRCA1* c.442-22_442-13del variant could be a founding variant in Chinese individuals of ancient southern Han descent (56).

Recently, a study including 21,216 unselected patients with BC and 6,434 healthy controls from 19 medical centers throughout 11 Chinese provinces identified 1,958 *BRAC1/2* variants through panel-based sequencing, of which 532 (27.2%) were pathogenic variants, and 858 (43.8%) were pathogenic variants of uncertain significance. The remaining 568 variants (29.0%) were benign. A total of 268 mutations in the *BRCA1* gene and 242 mutations in *BRCA2* were found in Asian patients with BC, most of which were meaningless mutations. Among these variants, researchers found 13 types of high-frequency lesions: p.Cys328fs, p.Asn704fs, p.Ser1862fs, and p.Ile1845fs in *BRCA1*; p.Ala938fs, p.Gln1037*, p.Ser1722fs, p. Tyr1894*, p.Leu1908fs, p.Glu2198fs, p.Ser2378*, p.Pro2802fs, and p.Thr3033fs in *BRCA2*. Eight of these variants have not been reported as high-frequency variants in Caucasians (57). Of note, single nucleotide polymorphisms (SNPs), a single nucleotide substitution at a specific position in the genome, may also contribute to *BRCA1* changes. For example, two pathogenic SNPs were found on the 11th exon of *BRCA1*, which may be related to early-onset BC in the Chinese population (58).

Although various *BRCA1*/*BRCA2* mutations are currently being investigated, variants of unknown significance that occur in *BRCA1*/*BRCA2* account for 12%–13% of the cases, the functions of which are still unclear. Therefore, more research is required to determine the clinical importance of variants of unknown significance in BC (59).

#### 3.3.2 *TP53*

The tumor suppressor gene *TP53*, located on chromosome 17p13.1, is the most commonly mutated gene in patients with cancer (60). The *TP53* gene encodes the cellular tumor antigen p53, an intracellular transcription factor that controls multiple tumor suppressive pathways (61, 62). *TP53* is defined as the “guardian of the genome” because of its role in conserving stability by preventing genetic mutations. The germline loss of *TP53* can quickly lead to the formation of spontaneous cancers (63, 64). Moreover, cancers with wild-type *TP53* predict a good prognosis (65), while those with mutant *TP53* predict a worse prognosis (66–68).

Notably, about 30% of the BCs have *TP53* mutations (60), most occurring in exons 5–8 (69). It has been reported that about 5% of the patients with BC with a positive FH and wild-type *BRCA1* and *BRCA2* carried a mutation in either *CHEK2* or *TP53* (70). In patients with Li-Fraumeni syndrome (LFS) with *TP53* mutations, the risk of developing BC under 45 years of age is 18–60 times higher than that of the general population (71). One study of 150 patients with familial and early-onset BC revealed that the deletion variant of *TP53* (643_660del18) appeared to have occurred only in the Chinese population (72).

#### 3.3.3 Phosphatase and Tensin Homolog (*PTEN*)

The *PTEN* gene is located on chromosome 10q23.31, which encodes phosphatidylinositol 3,4,5-triphosphate 3-phosphatase. This has lipid phosphatase activity and works antagonistically to the PI3K signaling pathway. It also negatively regulates the mitogen-activated protein kinase (MAPK) pathway through its protein phosphatase activity (73). *PTEN* mutation is also associated with Cowden syndrome, an autosomal dominant genetic disease that increases the lifetime risk of BC. In *PTEN* variant carriers, the lifetime risk of BC is estimated to be 67%–85% (74).

#### 3.3.4 *PALB2*/*FANCN*

*PALB2* is located on chromosome 16p12.2 and encodes a protein that interacts with *BRCA2* as a functional partner. Thus, *PALB2* affects the nuclear localization and stability of *BRCA2* and can also act as a bridge between *BRCA1* and *BRCA2* (38, 75). The biallelic *PALB2* mutation causes a new Fanconi anemia subtype, FA-N, also called *FANCN* (76, 77). Recent studies have found that germline mutations of *PALB2* exist in families with BC, indicating that *PALB2* may be a tumor suppressor for FBC (78, 79). Moreover, individuals with *PALB2* mutations have a 2.3 times higher risk of developing BC (43). One study conducted in Finland showed that *PALB2* c.1592delT is a founder variant, which causes truncated protein products with functional defects that cannot support *BRCA2* to complete DNA repair. As such, females with this *PALB2* mutation have a four times higher risk of developing BC (80). By age 50, the cumulative risk of BC in women with such mutations is estimated to be 14%, and by age 70, it increases to 35% (81). Zhang et al. also identified three harmful variants (c.3271delC, c.103C>T, and c.3035C>T) of *PALB2* in 305 cases of FBC in China, and the mutation rates were all 0.33% (82). In addition, studies have found that women with *PALB2* mutations from families with a history of BC have a greater risk of BC than those with no FH of BC (83).

#### 3.3.5 E-Cadherin (*CDH1*)

The *CDH1* gene is located on chromosome 16q22.1 and encodes the tumor suppressor, E-cadherin (a transmembrane calcium-dependent protein involved in cell-cell adhesion) (84–87). *CDH1* mutation is closely related to lobular breast cancer, and the lifetime risk of developing BC in those with a *CDH1* mutation is approximately 39%. Moreover, somatic and epigenetic changes in the *CDH1* gene are frequently detected in sporadic tumors, including BC, and are associated with worse survival rates (88, 89). Pathogenic mutations of *CDH1* are also the leading cause of hereditary diffuse gastric cancer (HDGC) (90), whose first clinical manifestation could be lobular breast cancer (91).

#### 3.3.6 Serine/Threonine Kinase 11/Liver Kinase B1 (*STK11/LKB1*)

The *STK11* gene is located on chromosome 19p13.3 and encodes a serine/threonine kinase that regulates many physiological processes, including energy metabolism and cell polarity (92). Most importantly, it is estimated that 32%–54% of the *STK11* gene mutation carriers under 60 years of age have a high risk of developing BC during their lifetime. The risk of developing BC for these carriers is 8% at the age of 40, increasing to 32% at the age of 60. Compared with the general population, the risk of BC in this subpopulation is seven times higher (93).

### 3.4 Genes With Moderate Penetrance

#### 3.4.1 Neurofibromatosis Type 1 (*NF1*)

The *NF1* gene is located on chromosome 17q11.2 and encodes neurofibromin, a cytoplasmic protein that regulates multiple critical signaling pathways, such as the Ras-cAMP pathway (94). Neurofibromin can increase the guanosine triphosphate (GTP) hydrolytic rate of Ras and, thus, plays a tumor-suppressive role by reducing Ras activity (95). Females under the age of 50 with *NF1* gene mutations have an up to five times higher risk of BC morbidity and mortality (96), and the risk was high among women under 40 years of age (97).

#### 3.4.2 Checkpoint Kinase 2 (*CHEK2*)

Cell cycle checkpoint kinase 2 (*CHEK2*) is involved in DNA damage and replication checkpoint responses and has been widely considered a BC-sensitive factor (98). The *CHEK2* gene is located on chromosome 22 and is critical for cell cycle regulation. The pluripotent kinase *CHEK2* is important for DNA damage response by causing cell cycle arrest or apoptosis. *CHEK2* phosphorylates the TP53 tumor suppressor protein and prevents its degradation during DNA damage, leading to G1 cell cycle arrest (99). In addition, *CHEK2* can induce cell apoptosis independent of TP53 by phosphorylating the tumor suppressor, promyelocytic leukemia protein (100). *CHEK2*.1100delC is a protein truncation variant first found in a family with LFS (101). Moreover, *CHEK2*.1100delC can be detected in 5.1% of the non-carriers of the *BRCA1* or *BRCA2* mutations with FH in northern Europe (102), and such mutation in females can increase the risk of developing BC by 2–3 times, and by 10 times in males (103, 104). A study based on a Chinese population with the *CHEK2* mutation in 74 patients with BC with FH and 50 control subjects identified that the missense variant of *CHEK2* in these cases was 1111C>T (His371Tyr) instead of *CHEK2*.1100delC, which implies that the *CHEK2*.1100delC variant was relatively rare in the Chinese population and that *CHEK2* c.1111C>T mutation might be related to the genetic susceptibility of BC (105). Another study screened the *CHEK2* coding sequence of 118 cases of Chinese FBC negative for *BRCA1* and *BRCA2* mutations and confirmed that the incidence of *CHEK2* c.1111C>T in FBC was higher than that in non-selective BC (4.24% vs. 1.76%) (106).

#### 3.4.3 Ataxia Telangiectasia Mutated (*ATM*)

The *ATM* gene is located on chromosome 11q, which encodes a phosphatase essential for substrate phosphorylation involved in DNA repair and cell cycle regulation (107). The *ATM* gene mutation rate in the general population is about 1%, and *ATM* heterozygotes have an increased risk of developing BC (108), especially women over 50 years of age (109). The lifetime risk of developing BC in patients with *ATM* monoallelic mutations is about 17%–52% (110, 111). A recent meta-analysis showed that the fully deleterious variants of *ATM* could cause a BC risk 2–4 times higher than that of the general population (112).

#### 3.4.4 Nibrin (*NBN*)

The *NBN* gene is located on chromosome 8q21.3 and encodes the Nibrin protein, responsible for interaction with DNA repair proteins involved in DNA double-strand break signaling (113, 114). Carriers of *NBN* monoallelic mutations have a significantly increased risk of BC, with an estimated odds ratio of 3.1 (115). Moreover, the truncated c.657del5 variant of *NBN* is also regarded as a high-risk factor for BC (116).

#### 3.4.5 *RAD51C * and *RAD51D*

Proteins encoded by the *RAD51* gene are important for DNA double-strand break repair. Seven *RAD51* paralogs have been identified in mammals, including *RAD51*, *RAD51B*, *RAD51C*, *RAD51D*, *XRCC2*, *XRCC3*, and *DMC1* (117). Based on the present knowledge, mutations in *RAD51C* and *RAD51D* are closely related to carcinogenesis (118, 119). For example, *RAD51C* and *RAD51D* gene mutations can be detected in patients with FBOC (120). One study from China conducted a genetic analysis of 273 patients with *BRCA1/2*-negative FBC and identified four previously unknown amino acid substitution variants in the *RAD51C* gene, the 4C>G (R2G) located in exon 1, 635G>A (R212H), and 644A>G (D215G) in exon 4, and 882G>C (Q294H) in exon 6. The R212H variant was unlikely to be pathogenic, as it only existed in healthy individuals. However, the R2G and D215G variants were suggested to be pathogenic to Chinese women after analysis using the SIFT, PolyPhen, and PMut algorithms (121). Moreover, a recent study showed that the protein-truncating variants in *RAD51C* and *RAD51D* are related to FBC. The estimated relative risks of BC associated with *RAD51C* and *RAD51D* mutations were 1.99 and 1.83, respectively. Therefore, they were classified into the moderate-risk category based on the current National Institute for Health and Care Excellence guidelines (57, 122).

### 3.5 Genes With Low Penetrance

#### 3.5.1 *MLH1, MSH2, MSH6,* and *PMS2*

*MLH1*, *MSH2*, *MSH6*, and *PMS2* encode DNA mismatch repair (MMR) proteins responsible for DNA mismatch repair (123, 124). Mutations in these genes can cause Lynch syndrome. Several studies have found that MMR gene mutations frequently exist in patients with BC (125), but the association between Lynch syndrome and BC is unclear (126).

With the widespread application of high-throughput sequencing, a large number of genes related to BC risk have been identified, such as *BAP1*, *PPM1D*, and *ABRAXAS1* (127, 128). However, their exact connection with BC and its penetrance remains unclear.

### 3.6 Gene Mutations in Chinese Patients with BC

Gene mutations related to BC are thought to vary among patients from different regions and races. Specifically, several gene mutations occurred mainly in Chinese populations (**Table 2**).

**Table 2:** Gene mutations in the Chinese population.

Population	Gene mutation	Finding
Chinese Han people (58)	*BRCA1*	Two pathogenic SNPs
BRCA1/2-negative Chinese FBOC (129)	*FANCC*	*FANCC* deleterious mutations
Chinese families from Singapore (56)	*BRCA1*	*BRCA1* c.442-22_442-13del variant
	*PALB2* and *RAD51D*	Common mutant genes
FBCs and early-onset BC from southern China (72)	P53	P53 643_660del18del variant
BCs or healthy people with a FH of BC from Henan, China (50)	*BRCA1*	*BRCA1* A3780G variant *BRCA1* A3113G variant *BRCA1* A3780G variant
BCs with a FH or early-onset BCs from Hunan, China (130)	*PTEN*	*PTEN* IVS4+109insTCTTA variant *PTEN* 225 A>C (Thr 160 Pro) variant (novel^1^) *PTEN* IVS5+13T>C variant (novel) *PTEN* rs121909229 G>A variant (Arg 130 Gln)
	*NBS1*	*NBS1* IVS6+43A>G variant (novel) *NBS1* IVS6+127A>G variant (novel) *NBS1* rs1805794 G>C variant (Glu 185 Gln)
BCs who had at least one first-degree relative affected from Shanghai, China (51)	*BRCA1*	*BRCA1* IVS17-1G>T variant (novel) *BRCA1* IVS21+1G>C) variant (novel) *BRCA1* 1100delAT variant *BRCA1* 5640delA variant
FBCs and/or early-onset BCs from eastern Shandong of China (52)	*BRCA2*	*BRCA2* 2001del TTAT variant (novel) *BRCA2* 4099C to T variant (novel) *BRCA2* 5873C to A variant (novel)
unrelated FBOCs from Eastern China (131)	*BRCA1*	LGR variants in *BRCA1* gene exon5-7dup (novel) exon13-14dup (novel) exon1-22del (novel)
Chinese BCs with a FH (105)	*CHEK2*	*CHEK2*.1100delC variant (not found) *CHEK2* 1111C>T (His371Tyr) variant
Chinese early-onset BC and/or affected relatives (132)	*RAD50* and *NBS1*	Not found
FBCs and high-risk women with a FH of BC from southern and central China (133)	*BRCA1, BRCA2, CHEK2, PALB2, ATM, BARD1, NBN, RAD51C, TP53, BRIP1* and *CDH1*	Detect mutations
	*MUTYH*	*MUTYH* c.892-2A > G variant (benign)
*BRCA1*/2-negative FBCs (134)	*DICER1*	Not found
Chinese FBCs and SBCs (82)	*PALB2*	*PALB2* c.3271delC variant (novel) *PALB2* c.103C>T variant *PALB2* c.3035C>T variant
Early-onset, bilateral or FBCs from Taiwan, China (53)	*BRCA1*	*BRCA1* c.5191C>A variant (novel) *BRCA1* c.1155C>T variant (benign)
sporadic and *BRCA1*/2-negative FBCs (135)	*GADD45A*	Not found
BCs with at least one first-degree relative affected with BC from Shanghai, Jinan, Qingdao, and Shenyang (54)	*BRCA1*	*BRCA1* 1100delAT variant *BRCA1* 5589del8 variant (novel)
Chinese *BRCA1*/2-negative FBCs (106)	*CHEK2*	*CHEK2* c.1111C>T (p.H371Y) variant
Chinese *BRCA1*/2-negative FBCs (121)	*RAD51C*	*RAD51C* 4C>G (R2G) variant (novel) *RAD51C* 644A>G (D215G) variant (novel) *RAD51C* 635G>A (R212H) variant (novel) *RAD51C* 882G>C (Q294H)) variant (novel)
Chinese FBCs (136)	Mitochondrial DNA (mtDNA)	Sequence variants within the mtDNA D-Loop region, particularly those in D310 segment

^1^Novel means this variant has been reported for the first time.

Generally, most mutations in *BRCA1/2* genes exist in Chinese populations; however, *ATM*, *CHEK2*, *PALB2*, and *BRIP1* have more pathogenic mutations among non- *BRCA1/2* carriers (137).. One study conducted a 27-gene panel analysis of 120 patients with BC and 120 high-risk women with first-degree or second-degree relatives. The study identified that 12 genes contained harmful mutations in the Chinese population, including *BRCA1*, *BRCA2*, *MUTYH*, *CHEK*, *PALB2*, *ATM*, *BARD1*, *NBN*, *RAD51C*, *TP53*, *BRIP1*, and *CDH1* (133).

One study conducted in Hunan, China, first reported two variants in the *PTEN* gene (225A> C (T160P) and IVS5 +13T> C) and two variants in the *NBS1* gene (IVS6 + 43A> G and IVS6 + 127A> G) in familial and early-onset BC in the study population (130). *FANCC*, which belongs to the Fanconi anemia complementation group, has been reported as a susceptibility gene for BC. It was found that there were harmful variants of *FANCC* in patients with FBOC in China, but its penetration and spectrum require further study (129). Other genes, such as *DICER1* (134), and growth arrest and DNA damage-induced 45 alpha (*GADD45A*) (135) have been reported as candidate susceptibility genes for FBC, but no mutations in these genes have yet been discovered to be harmful among the Chinese population.

### 3.7 BC-Related Genetic Syndromes

The above-mentioned BC susceptibility genes are active in several genetic syndromes related to BC. Due to the existence of mutant genes, patients with a family history of these genetic syndromes have a higher risk of BC than the general population. These BCs show familial aggregation, i.e., FBC.

Next, we introduce the genetic syndromes that have been confirmed to be associated with an increased risk of BC (**Table 3**).

**Table 3:** BC-related Genetic Syndromes.

Genetic Syndromes	Relate genes	Locus
Hereditary Breast and Ovarian Cancer Syndrome (HBOC) (138)	*BRCA1*	17q21.31
	*BRCA2*	13q13.1
Li-Fraumeni Syndrome (139)	*TP53*	17p13.1
Ataxia Telangiectasia (140)	*ATM*	11q22.3
Cowden Syndrome (141)	*PTEN*	10q23.31
Peutz-Jeghers Syndrome (142)	*STK11*	19p13.3
Hereditary Diffuse Gastric Cancer syndrome (HDGC) (143)	*CDH1*	16q22.1

#### 3.7.1 Hereditary Breast and Ovarian Cancer (HBOC)

HBOC is an inherited disorder that was first reported in the 1970s (144). The clinical characteristics of HBOC include young age of onset, multiple family members with BC or OC, or both, or one family member with both BC and OC, or bilateral BC (145). The most common genetic changes associated with HBOC are mutations in the *BRCA1* and *BRCA2* genes (30). Approximately 60% of the typical families with HBOC harbor BRCA mutations (70). In China, this proportion was approximately 15.8% (146). Finally, because BRCA gene mutations are common in FBC, some patients with HBOC exhibit similar symptoms as patients with FBC.

#### 3.7.2 Li-Fraumeni Syndrome (LFS)

LFS was first discovered in 1969 as an autosomal dominant malignant tumor syndrome caused by a germline mutation in the *TP53* tumor suppressor gene. The *TP53* gene mutation can be detected in approximately 50%–70% of the LFS cases, which is much higher than its prevalence of 1% in BC cases (147, 148). Furthermore, patients with LFS may suffer from multiple cancers, including BC, brain tumors, soft tissue sarcoma, leukemia, osteosarcoma, adrenal cortical malignancies, and broncho-alveolar lung cancer. The lifetime risk of developing BC in patients with LFS is estimated to be 25%–79% (149, 150). Some patients with BC of LFS show familial aggregation. Therefore, the NCCN guidelines recommend that women with *TP53* pathogenic variant/likely pathogenic variant undergo a clinical breast examination every 6–12 months beginning at the age of 20 and a breast magnetic resonance imaging (MRI) with contrast screening every year from 20–75 years (151).

#### 3.7.3 Hereditary Diffuse Gastric Cancer (HDGC)

HDGC is an autosomal dominant genetic disease caused by a *CDH1* mutation. Patients with HDGC are susceptible to lobular breast cancer (LBC). The cumulative risk of LBC in women with *CDH1* mutations is estimated to be 39%–52% by age 80 (143, 152, 153).

#### 3.7.4 Ataxia Telangiectasia

Ataxia telangiectasia is an autosomal recessive genetic disease that is closely related to *ATM* mutations. The clinical manifestations include eyelid telangiectasia, cerebellar ataxia, and immunodeficiency (140). Some studies have shown that heterozygous carriers of *ATM* mutations have an increased risk of BC. A meta-analysis including three cohort studies of relatives of patients with ataxia telangiectasia estimated that the relative risk of BC in patients with ataxia telangiectasia is approximately 18% at 80 years of age (112). Moreover, if one has a history of radiation exposure, the risk increases further (154).

#### 3.7.5 Cowden Syndrome

Cowden syndrome is an autosomal dominant genetic disease, which is clinically characterized by hamartoma-like lesions, pathognomonic skin lesions, benign breast disease, early-onset BC, and thyroid cancer. This disease is closely related to the *PTEN*/*MMAC1*/*TEP1* gene mutations. Furthermore, approximately 75% of the female patients with Cowden syndrome harbor multiple benign breast lesions, such as fibroadenoma, cystic lesions, and ductal hyperplasia (155). A French study estimated that the cumulative BC risk in patients with Cowden syndrome was between 25 and 85%, and the cumulative incidence of BC by the age of 70 was 77% (95% CI: 59%–91%) (156).

#### 3.7.6 Peutz-Jeghers Syndrome

Peutz-Jeghers syndrome is a rare autosomal dominant genetic disease caused by mutations in the *STK11* gene. The clinical manifestations of Peutz-Jeghers syndrome include hamartoma-like polyps in the gastrointestinal tract, melanin deposition in the skin and mucous membranes, pancreatic cancer, and mucocutaneous periorificial lentiginosis. The lifetime risk of patients with Peutz-Jeghers syndrome developing BC is 24%–54%, and the average age of onset is approximately 39 years (157, 158). The NCCN guidelines recommend that carriers of *STK11* gene mutations undergo clinical breast examinations every 6 months and annual mammography and breast MRI examinations from the age of 25 (159).

### 3.8 Screening Strategies for FBC

**Figure 6:**
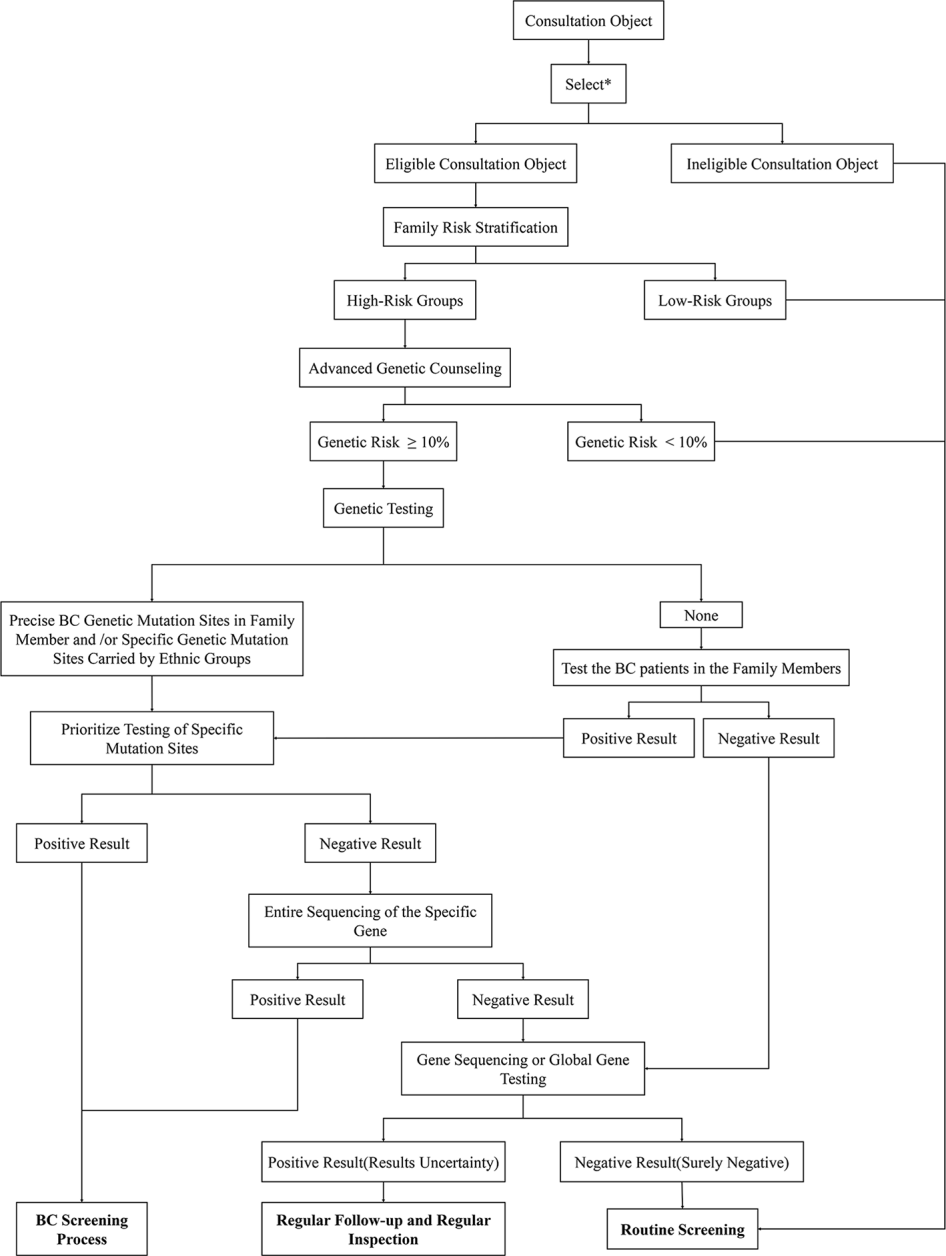
Genetic counseling strategies for high-risk BCs in China. First, identify eligible consultation participants through the listed criteria. Then use risk stratification tools to screen high-risk groups from the selected subjects. The lifetime risk of BC of these participants will be calculated through the risk assessment model in preliminary genetic counseling. High-risk subjects with a disease risk greater than 10% will be further subjected to follow-up genetic testing. Genetic testing gives priority to the detection of high-risk mutant gene sites in individuals of a family or Chinese populations. If obvious abnormalities are found, then specific mutation sites can be considered, which should undergo the BC screening process; if there are no abnormal findings, the entire sequence can be considered. In addition, the positive subjects receive BC screening while the negative participants receive whole genome sequencing. The final genetic test results need to be compared with the genetic test results of patients with BC in the family. If the mutant gene is the same, the consultation subject is confirmed to be in the high-risk group; if the results are inconsistent, the subject will not be listed as high-risk for the time being. For those whose family members have not been tested for genetic mutations, the first step is to detect the mutated gene sites in patients with BC. If the family member cannot be tested or the test result is negative, whole-genome sequencing can be performed. If there are clear mutation sites in the family members with BC, priority will be given to monitoring these gene sites. The subsequent detection steps are the same as those described above. Result analysis: 1. Positive Result (The consultation participant showed clear mutation genes that were consistent with those of a family member or ethnic group): Corresponding measures can be taken to actively intervene in the clinic; 2. Uncertainty negative (no obvious abnormality was found in the gene testing, but whole genome sequencing was not performed due to other factors): regular clinical follow-up; 3. Results Uncertainty (the patient found clear mutation genes but inconsistent with family): Regular clinical follow-up; 4. Surely negative (no abnormality was found after all inspections were completed): Routine examination. *The selection criteria are shown in **Figure 7**.

FBC is characterized by familial aggregation, and family members of patients with FBC have a higher lifetime risk of disease than the general population (8). Generally, the screening processes for high-risk groups of FBC are as follows: professional genetic counselors use screening tools to identify potentially diseased members of the family; women who have a positive screening result receive genetic counseling to decide whether to perform advanced genetic counseling or *BRCA* genetic testing; and finally, early monitoring and physical examination of these high-risk groups are carried out to achieve early detection and treatment (160). This set of procedures is utilized in some countries (161); however, due to the differing FBC gene mutations among ethnic and geographical groups, when applied to the Chinese population, this procedure needs to be modified to meet the differing needs of this specific population (**Figure 6**).

#### 3.8.1 Genetic Counseling

Genetic counseling is a counseling process for relatives who have genetic diseases or are at risk of infection to provide disease occurrence and early detection or prophylactic intervention methods. Genetic counseling can prevent genetic diseases and provide insight regarding reproductive options and should be conducted by a well-trained professional counselor. The professional counselor conducts family investigation and analysis by evaluating the proband in the family and estimating the possibility of the disease in the offspring.

**Figure 7:**
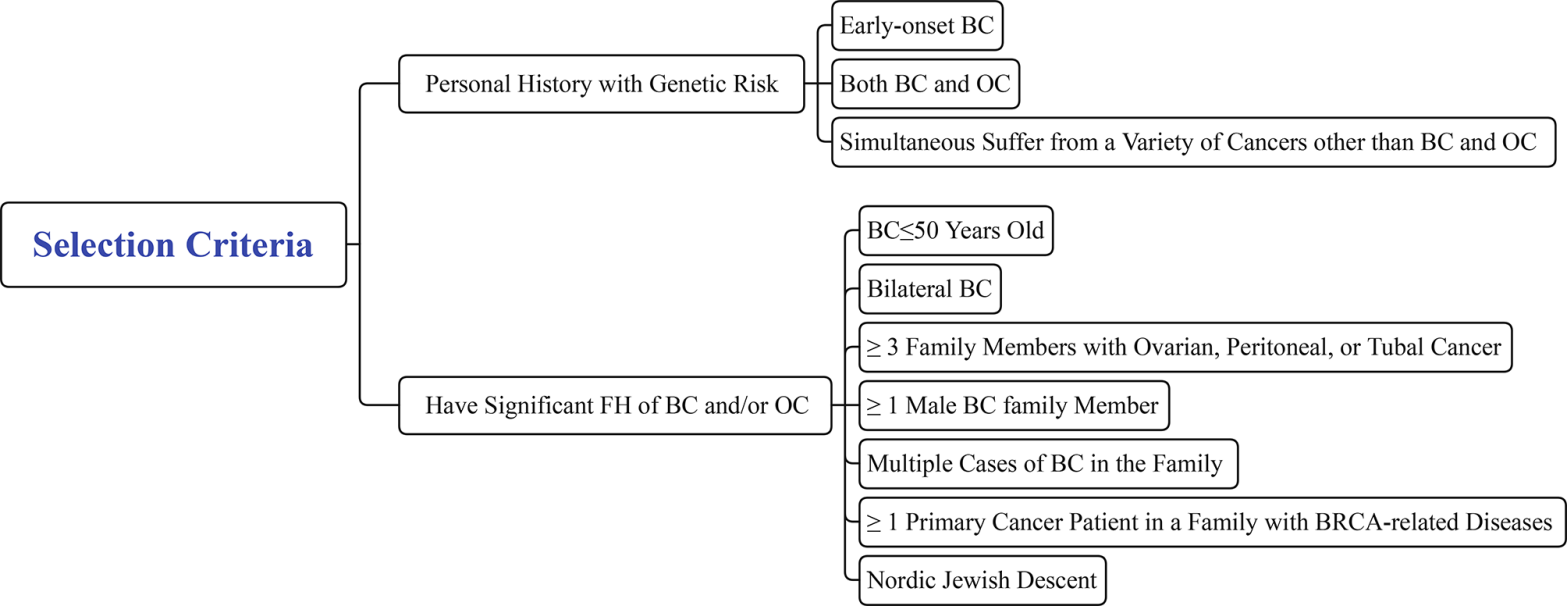
Selection criteria for consulting participants.

Genetic counseling for healthy family members of FBC is an important step in the screening process for high-risk populations and should be performed before blood is drawn to collect DNA to identify pathogenic mutations. However, not all individuals are required to receive genetic counseling. Eligible participants should meet one of the following criteria: 1. Personal medical history with genetic risk of i) early-onset BC (≤35 years old); ii) both BC and OC; iii) simultaneous cancers other than BC and OC; and 2. Significant FH of BC/OC: i) BC ≤50 years old; ii) bilateral BC; iii) at least three family members with ovarian, peritoneal, or tubal cancer; iv) at least one male family member with BC; v) multiple cases of BC in the family; vi) at least one primary cancer patient in the family with *BRCA*-related diseases; and vii) Nordic Jewish descent (**Figure 7**) (4, 160).

During genetic counseling, genetic counselors can use family risk stratification tools, such as the Manchester Scoring System (MSS), Family History Screen-7, Pedigree Assessment Tool, Referral Screening Tool, and Ontario Family History Assessment Tool (Ontario-FHAT) to distinguish the participants who need follow-up consultation. Healthy populations with a high risk of BC need advanced genetic counseling or *BRCA* gene mutation testing (162, 163). For others with a low risk of BC, routine screening strategies are recommended (161). Following the BC screening strategy for the Chinese population (2021 version), the low risk population undergoes X-ray or B-ultrasound examinations every 1–2 years, while high-risk groups undergo annual X-ray and B-ultrasound examinations and breast MRI when necessary. Patients that fall between low and high risk undergo X-ray and B-ultrasound examinations every 1–2 years (164).

#### 3.8.2 Genetic Testing

Genetic testing is conducive to the early detection of high-risk groups of BC, providing preventive measures, and improving prognosis (165). Currently, only two genes (*BRCA1* and *BRCA2*) are routinely used for BC genetic testing, and the test subjects are usually limited to women with an FH of BC and OC. However, given that genetic testing is relatively expensive and may cause adverse socio-psychological effects, testing should target individuals that most likely have gene mutations (166, 167). For example, *BRCA1/2* testing is only provided to individuals with a mutation probability greater than or equal to 10% in many regions such as British (168). Therefore, advanced genetic counseling or models should be used to predict the likelihood of *BRCA1* and *BRCA2* mutations before performing genetic testing. When a patient qualifies as a test participant, they should fully understand the advantages and disadvantages of genetic testing and provide fully informed and written consent before genetic testing is possible (5).

It is generally recommended that female test subjects younger than 18 years of age undergo *BRCA* genetic testing. The focus of mutation analysis depends on the FH. If there are family members with BC with precise genetic mutation sites or ethnic groups carrying specific genetic mutation sites, these sites can be tested first (169). If the test subject does not have these characteristics, the entire specific gene sequence must be tested to exclude other site mutations. It is worth noting that women who are clinically negative for *BRCA* mutations are also at risk for BC (170). Walsh et al. found that among high-risk American families for BC and whose *BRCA1* and *BRCA2* gene test results are negative, approximately 12% still carry genetic mutations in *BRCA1*, *BRCA2*, *CHEK2*, *TP53*, and *PTEN* (70). These false negatives can easily lead to a missed diagnosis in high-risk groups. In addition, most of the gene mutations in FBC are unrelated to *BRCA1*, such as *ATM*, *CHEK2*, and *BARD1*, in the homologous recombination pathway (171). Further studies have shown that the truncated variants of *ATM* and *CHEK2* are more closely associated with estrogen receptor (ER)-positive BC, while *BARD1*, *BRCA1*, *BRCA2*, *PALB2*, *RAD51C*, and *RAD51D* are more closely related to ER-negative BC (29). Therefore, it is believed that both sequencing and global screening for rearrangements should be performed for women with high risk for BC who are *BRCA1/2* negative. For families with only negative *BRCA1/2* sequencing test results, multiplex ligation-dependent probe amplification testing can be performed.

If the result of genetic testing is positive, that indicates the detected mutant gene is the same as the mutant gene of the BC patient in the family; therefore, the test subject needs to continue the screening process. Subsequent BC screening includes regular breast self-examination and imaging examinations. If the BC patient is not a mutation carrier, the test subject will temporarily not be listed as a high-risk patient. Those participants whose tests are all negative will temporarily not be classified as high-risk groups, and the general population screening process is recommended (161).

With the development of genetic testing, many new testing methods have emerged, such as multigene panel testing, which can detect multiple gene loci simultaneously. In a study of 35,000 women with BC, the 25-gene panel showed mutations most commonly in *BRCA1*, *BRCA2*, *CHEK2*, *ATM*, and *PALB2* (172). Moreover, NCCN Guidelines 2017 stated that if more than one gene can explain hereditary cancer, polygenic testing may be required to be more effective and cost-effective. A recent study also demonstrated that population-based multigene panel testing is more cost-effective than individual *BRCA1/BRCA2* testing (173). Moreover, a current study in China showed that multigene panel testing could increase the mutation detection rate in patients with a high risk of BC (137). Thus, although multigene panel testing still has shortcomings for practical applications (174), it may become an important technology in future FBC screening strategies.

From a technical point of view, it is practical to accurately detect *BRCA1/2* mutations and other susceptible gene mutations in China. However, there is currently no uniform standard for the hot spots of FBC mutant genes in the Chinese population. Moreover, there is no uniform standard for genetic testing. The shortage of professionals to provide genetic counseling results in the imperfect prevention of uncertain genetic mutation carriers. Therefore, establishing an entirely professional team and standardized genetic counseling processes for FBC prevention and treatment are urgently needed.

#### 3.8.3 Predictive Model

Only 5%–10% of the BC susceptibility gene carriers will eventually develop BC due to gene variations. Predicting the risk of BC in individuals with FH is helpful for the clinical prevention and treatment of FBC. At present, many clinical models that combine the patient’s personal history, FH, and other factors to assess the risk of BC have been proposed (175), although no standardized prediction method has been widely accepted.

#### 3.8.4 Gail Model (1989)

The Gail model is a statistical analysis model based on case-control data, which integrates the risk factors of BC, including BC history, age, age at menarche, age at first birth, number of first-degree relatives with BC, breast biopsy results, and race. Gail-1 in 1989 was initially used to predict the risk of invasive BC and carcinoma *in situ* in white women who underwent mammography each year (176). Gail et al. modified Gail-2 in 1990 to improve its predictive power (175). Since then, the Gail model has been validated in many different populations (white, African American, Hispanic, Asian American, American Indian, and Alaska Natives).

#### 3.8.5 Claus Model (1994)

The Claus model estimates the lifetime risk of BC based on the FH of first- and second-degree relatives with BC and OC (177). The reference parameters included the age of onset of the paternal and maternal lines and information on cancer history (178).

#### 3.8.6 Penn Model (1997)

The Couch (Penn I) model was initially used to predict the probability of *BRCA1* mutations in 169 families with BC. The Penn II model is based on logistic regression analysis and integrates specific clinical features (such as bilateral BC) to predict the possibility of *BRCA1*/2 mutations in individuals or families. Studies have shown that the prediction accuracy of the Penn II model is higher than that of the Penn I and Myriad II models (179).

#### 3.8.7 BRCAPRO Model (1998)

The BRCAPRO model is based on the Bayes theorem, the prevalence of BC and OC in first- and second-degree relatives, and the age of onset of the disease in family members to screen *BRCA1*/2 gene mutation carriers. The model was developed into a computer software in 2002 (180, 181). At present, the model is continuously updated, and its feasibility has been confirmed (182).

#### 3.8.8 Myriad Model (2002)

The Myriad model is based on 7,461 samples tested for *BRCA1*/2 mutations and 2,539 samples of detected mutations in three descendants of Ashkenazi Jewish ancestry (including FH, age of onset of FBOC, and presence of invasive cancer) to establish a model to predict the possibility of carrying mutations. The model has been publicly released at present, and the sample size is being expanded for research and updates (183).

#### 3.8.9 BOADICEA Model (2004)

The BOADICEA model was developed based on the complex isolation analysis of the occurrence of BC and OC in the combined data of 1,484 BC cases and 156 multi-case families (184). This model not only allows for simultaneous effects of both *BRCA1* and *BRCA2* but also allows for the effects of genetic modifiers and the multiplier effect of low penetrance genes on BC risk (185).

#### 3.8.10 Manchester Scoring System (2004)

Evans et al. used whole-gene screening technology to screen the DNA samples of 422 non-Jewish patients with a history of BC/OC for *BRCA1* mutations and performed *BRCA2* screening for 318 subsets. After combining the screening results and FH, a simple scoring system, the Manchester scoring system, was designed to predict pathogenic mutations (186). In 2009 and 2017, MSS2 and MSS3, respectively, were released, where the pathological weight was considered in the scoring system, and the corresponding score was adjusted (187, 188). For subjects in Northwest England, a total MSS3 score of 15–19 is equal to the 10% threshold, and a score of 20 points is equal to the 20% threshold, but the threshold may need to be adjusted when applied to other populations.

## 4 Discussion

BC is currently the most common cancer among women worldwide. The incidence of BC in 2020 is the highest while the mortality rate is only fifth in the world, highlighting the need to prevent and treat BC. Compared with SBC, the cause of FBC seems to be clearer. Many studies have discovered multiple gene mutations related to FBC, such as *BRCA1*, *ATM*, and *CHEK2*. Scholars divide these gene mutations into three categories based on the RR. As the research progressed, some genes that were originally considered to be moderate risk were reclassified into the high penetrance category, such as *TP53*. Some of these gene mutations also exist in the Chinese BC population, while some specific gene variants, such as *PALB2* c.3271delC, are relatively unique to the Chinese population. In summary, we introduced 16 genes that have been identified to be closely associated with FBC. Accordingly, we described these genes in detail based on their different trajectories. Moreover, genetic syndromes associated with FBC have also been discussed with the corresponding risk of disease, such as HBOC, LFS, and HDGC. The NCCN guidelines also recommend corresponding screening strategies for these genetic diseases. However, such guidelines for the Chinese population have not yet been released. Considering the heterogeneity of BC, we listed the hotspot mutation genes and their loci in the Chinese FBC population and then proposed a screening flowchart for high-risk Chinese populations for FBC based on existing screening strategies.

At present, there is no standardized process for the diagnosis and treatment of FBC in China. High-risk groups can be screened out only through FH screening. After genetic counseling, some people are selected for corresponding genetic testing based on economic conditions as well as the counseling results, and the genetic testing results are combined to provide complementary preventive treatments. Although many predictive models have been established to predict disease risk, and some of them seem to be applicable to the Chinese population, there is currently no professional model designed specifically for the Chinese population. The lack of suitable predictive models and professional genetic counseling has led to the lack of standardization of early screening of FBC high-risk populations in China. An FBC screening strategy based on the risk of mutant genes specifically for the Chinese population is therefore urgently needed. This strategy can help in the early identification of high-risk patients among the families of patients with BC with FH in the clinic and provide a framework for subsequent in-depth therapeutic interventions and research. Due to the vast territory of China, the characteristics of BC in different areas or ethnic groups vary. It is difficult to collect the BC characteristics of the entire country, and further studies that can provide more objective evidence are required.

This study has several limitations. First, it lacks a systematic quantitative analysis, and there is a certain selection bias in the selection of the cited documents in this review, which may lead to bias in the research results and conclusions. In addition, the study of the Chinese population only selected populations in a particular region. Whether the results apply to the entire Chinese population remains to be confirmed.

In conclusion, early detection of FBC is a critical step in its treatment. FH, related mutant genes, and genetic syndromes provide a solid foundation for genetic counseling. For the Chinese population, different screening strategies need to be adopted based on unique genetic information.

## Author Contributions

LS wrote the first draft of the manuscript. All authors contributed to the article and approved the submitted version.

## Funding

This work was supported by the Zhejiang Provincial Natural Science Foundation of China (grant no. Y19H160283).

## Conflict of Interest

The authors declare that the research was conducted in the absence of any commercial or financial relationships that could be construed as a potential conflict of interest.

## Publisher’s Note

All claims expressed in this article are solely those of the authors and do not necessarily represent those of their affiliated organizations, or those of the publisher, the editors and the reviewers. Any product that may be evaluated in this article, or claim that may be made by its manufacturer, is not guaranteed or endorsed by the publisher.
